# Strategies for Piloting a Breast Health Promotion Program in the Chinese-Australian Population

**Published:** 2011-12-15

**Authors:** Fung Kuen Koo, Cannas Kwok, Kate White, Natalie D'Abrew, Jessica K Roydhouse

**Affiliations:** Sydney Nursing School; Cancer Nursing Research Unit, Sydney Nursing School, The University of Sydney and Sydney Cancer Centre, Royal Prince Alfred Hospital, Sydney, Australia; Cancer Nursing Research Unit, Sydney Nursing School, The University of Sydney and Sydney Cancer Centre, Royal Prince Alfred Hospital, Sydney, Australia; Cancer Nursing Research Unit, Sydney Nursing School, The University of Sydney and Sydney Cancer Centre, Royal Prince Alfred Hospital, Sydney, Australia; Cancer Nursing Research Unit, Sydney Nursing School, The University of Sydney and Sydney Cancer Centre, Royal Prince Alfred Hospital, Sydney, Australia

## Abstract

In Australia, women from non–English-speaking backgrounds participate less frequently in breast cancer screening than English-speaking women, and Chinese immigrant women are 50% less likely to participate in breast examinations than Australian-born women. Chinese-born Australians comprise 10% of the overseas-born Australian population, and the immigrant Chinese population in Australia is rapidly increasing. We report on the strategies used in a pilot breast health promotion program, *Living with Healthy Breasts*, aimed at Cantonese-speaking adult immigrant women in Sydney, Australia. The program consisted of a 1-day education session and a 2-hour follow-up session. We used 5 types of strategies commonly used for cultural targeting (peripheral, evidential, sociocultural, linguistic, and constituent-involving) in a framework of traditional Chinese philosophies (Confucianism, Taoism, and Buddhism) to deliver breast health messages to Chinese-Australian immigrant women. Creating the program's content and materials required careful consideration of color (pink to indicate femininity and love), symbols (peach blossoms to imply longevity), word choice (avoidance of the word *death*), location and timing (held in a Chinese restaurant a few months after the Chinese New Year), communication patterns (the use of metaphors and cartoons for discussing health-related matters), and concern for modesty (emphasizing that all presenters and team members were female) to maximize cultural relevance. Using these strategies may be beneficial for designing and implementing breast cancer prevention programs in Cantonese-speaking Chinese immigrant communities.

## Introduction

Cultural beliefs may be barriers to attendance at breast cancer screening services by women of Chinese ancestry living in Western societies (1). These beliefs include a perception of discussion about breasts as inappropriate and immodest and the view that breast cancer is a contagious disease that primarily affects Western women (2). Furthermore, the Buddhist concept of *karma* indicates that there are cultural taboos against talking about disease and death (3), akin to the idea of "speak of the devil and he will appear" (4). Fatalistic attitudes may also inhibit Chinese women's participation in cancer screening services (5,6).

Because cultural beliefs can influence health behaviors, the importance and effectiveness of culturally sensitive health promotion programs have long been recognized, particularly in multicultural societies, in which materials and programs that address the needs of the majority ethnic/cultural group are not necessarily relevant for all cultural groups. Such programs have produced promising results in Asian communities in Western countries with regard to improved breast cancer screening knowledge, intentions, and self-reported practice. For example, an intervention using socioculturally tailored breast health articles in community newspapers improved knowledge and self-reported practice of breast examination among South Asian women in Canada (7), and a culturally tailored video improved Chinese-American women's knowledge of and intentions regarding mammography (8).

In Australia, efforts have been made to promote breast cancer screening among women from minority cultural groups, for instance by providing printed materials in languages other than English. However, cultural factors can affect how health promotion messages are received, so simply translating health messages without accounting for cultural differences is unlikely to be effective (9). Chinese-born Australians are the largest group of immigrants from non-English-speaking backgrounds in Australia, comprising 10% of the overseas-born population (10). From 1981 to 2008, the Chinese population in Australia has increased substantially, from 25,200 to 313,000 (11). Sydney has the largest Chinese immigrant population of all Australian capital cities. People of Chinese ancestry comprise 7.1% of the population in Sydney, and the most commonly spoken dialect for Chinese immigrants in Sydney is Cantonese (52%) (12).

National data on the rates of mammography screening among each ethnic group are not collected in Australia. However, in Australia, women from non–English-speaking backgrounds participate less frequently (4%) in breast cancer screening than do English-speaking women (58%) (13), and Chinese immigrant women are 50% less likely to undergo breast examinations than are Australian-born women (14). In light of the rapidly increasing Chinese immigrant population in Australia and Chinese women's lower likelihood of participation in mammography screening (2), the need to design culturally appropriate breast health promotion programs for this population is urgent.

Reports of strategies used to target programs more effectively are of use to practitioners who are designing such programs. Many such reports exist, primarily describing programs in North American settings. Meade et al (15,16) describe their experience in designing a breast cancer program for Haitian immigrants in the United States, and other reports describe the development of culturally appropriate breast cancer resources for Chinese immigrant and minority populations (8,17) and intervention programs for Hmong immigrants (18). Reports from Australian settings have been limited, leaving a gap in the literature. To increase awareness of breast cancer and promote screening in the Chinese-Australian community, we developed the *Living with Healthy Breasts* (LWHB) program. To the best of our knowledge, this program is the first of its kind in Sydney, Australia. Guided by a conceptual framework of Chinese philosophies, we employed 5 types of strategies commonly used for designing culturally appropriate health promotion programs (peripheral, evidential, sociocultural, linguistic, and constituent-involving [9]) to target health information to our participants on the basis of cultural constructs (19).

We explain how we employed these strategies to deliver breast health messages to Cantonese-speaking Chinese-Australian women and encouraged the women to disseminate these messages in their communities. An assessment of the program and the participants' experiences is reported elsewhere (20); the focus of this article is the strategies used to make the program culturally and linguistically appropriate for the participants.

## The Conceptual Framework: Chinese Philosophies

Philosophies such as Confucianism, Taoism, and Buddhism should be considered when designing a culturally appropriate breast health promotion program for Chinese women because these philosophies serve as a foundation for concepts of health and illness, health promotion, and health-seeking behavior (21). In Confucianism, emphasis is placed on maintaining family harmony and on maintaining the family's patriarchy and lineal structure. Being an ideal person involves good-heartedness and responsibility and respect for elders (22). Taoism is essentially a philosophy of "let it be"; it emphasizes the need to live in harmony with nature and teaches that humans should follow the rhythms of heaven and earth to achieve a state of balanced tranquility (21). A central focus of the Chinese Buddhist doctrine is "cause and effect," sometimes called *karma*, which is the principle that encourages people to do "good" and "right" to receive "good" in return in the lives lived after this life (23).

## Program Design and Development

The LWHB program protocol was approved by the Human Research Ethics Committee of the University of Sydney. LWHB was conducted as a 1-day educational program with a 2-hour follow-up session 6 weeks later. The 1-day educational program had 4 sessions: 1) Promoting Breast Awareness, which focused on issues of breast health and screening; 2) Breast Cancer and Its Early Detection, which covered misconceptions and myths about breast cancer and breast health; 3) Communication Skills, which focused on training participants to be "breast health advocates" who could disseminate the breast health message in their communities; and 4) Introduction to the Information Kit, which introduced participants to a take-home information kit with materials to support their activities as breast health advocates. Six weeks after the educational training, participants had an opportunity during a 2-hour follow-up session to consolidate the information from the 1-day educational program and address any questions.

## Peripheral Strategies: Providing Culturally Appropriate Information Materials

Peripheral strategies use familiar symbols and images to make presentation and materials appealing and relevant (9). Therefore, we used culturally relevant colors and symbols during the education sessions and in the information kit (Figure). Furthermore, we avoided using words and numbers with negative associations and carefully considered the location, timing, and use of images.

**Figure.**
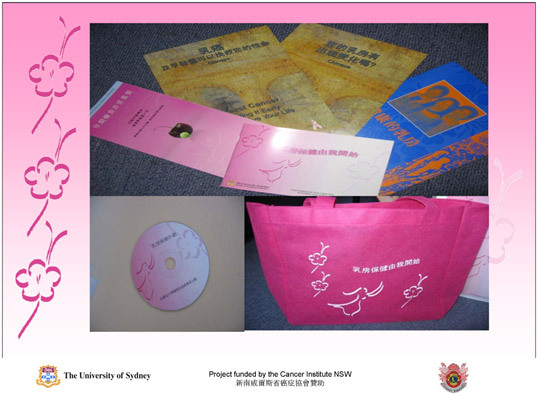
Each participant was given an information kit that contained an information booklet, a CD, and a pink woven bag on which "Living With Healthy Breasts" was printed.

Because pink indicates femininity and love in Chinese culture (24), we designed all printed program materials and the bag for the information kit with a pink color theme and avoided colors associated with bad luck, such as the color black (25,26). We used a peach blossom logo because peach blossom flowers imply longevity, growth, prosperity, and auspicious fortune (27); likewise, depictions of crosses, hospitals, and skeletons were avoided because they are associated with sickness. By using these positive colors and symbols and avoiding negative colors and symbols, we aimed to convey a feeling of love and good fortune and increase the acceptability of the program content, although it covered the sensitive and frequently taboo topics of breast cancer and breast screening.

We also avoided using words and numbers in presentation and printed materials that were associated with bad luck, bad fortune, or death (28,29). Special precautions were taken during the training session and follow-up session to avoid transgressing cultural taboos, such as mentioning sickness or dying, when asking hypothetical questions. We avoided directly mentioning the word *death* because, according to *karma*, the mention of illness or death can lead to its occurrence (3). Accordingly, we titled the program "Living with Healthy Breasts," which conveyed a positive feeling and excluded any reference to breast cancer, per recommendations from members of several Chinese community organizations. We also excluded from program materials the "bad luck" numerals of 4, 7, 24, and 44, for which the pronunciation is similar to the word death (28,30,31).

Location and timing were considerations in our efforts to maintain harmony. Therefore, we held the educational program and follow-up session in a Chinese restaurant located in Sydney's Chinatown, a familiar and easily accessible environment for participants. The program was delivered in June, a few months after the Chinese New Year, in recognition of the belief that seeking health care or talking about diseases and death before or during festive seasons is bad luck and will eventually cause illness (32).

Finally, we provided participants with an information kit that used cartoon images of Asian women and avoided images of women before or after breast surgery, which could be perceived as bringing bad fortune. The kit also contained the presentations from the education session, a mini-booklet that summarized the program in simple Cantonese language, and a model breast to demonstrate what a breast lump feels like. The model breast provided an easy way for the presenters and the participants to discuss this sensitive topic comfortably.

## Evidential Strategies: Raising Participant Awareness

Evidential strategies help encourage behavioral change by showing members of a particular group how a certain health issue affects the entire group (9). These strategies are grounded in the precaution adoption model, a multistage model that proceeds from lack of awareness about an issue to action on the issue (and maintenance, if appropriate) (33). Although evidential strategies are often operationalized by presenting data specific to a certain population (9), more nuanced approaches may be required. For example, evidential strategies for communicating about cancer with African Americans should avoid using data in which whites are the sole comparator and should use family examples to make statistics more relevant (ie, "social math") (34).

Because Chinese culture emphasizes social harmony and respect (4,22) and cancer is a sensitive topic (35), we did not begin the program with a discussion of breast cancer. Instead, we first presented tips for a healthy lifestyle and then demonstrated how these tips related to breast health. However, rather than immediately beginning a discussion on breast cancer, we referred to some common conditions, such as different breast sizes and retracted nipples. We then gradually introduced topics such as the incidence of breast cancer in Australia and the risk of breast cancer for Chinese immigrant women. We used images of women with Asian features to represent the ratio of the incidence and the risk of breast cancer. During the presentation, real scenarios involving Chinese-Australian women were shared with the participants. The first 2 authors (F.K.K, C.K.), who are of Chinese ancestry, shared their family members' experiences with breast cancer and discussed their own breast screening practices. Because some literature indicates that Chinese women perceive breast cancer as a disease specific to Western women (2), we emphasized that breast cancer is also the most common cancer among Chinese women. This point was also underscored by the aforementioned discussion of breast cancer experiences in Chinese families. To highlight the importance of breast cancer screening, we advised women that factors such as changes in the external environment, diet, and fertility can lead to an increased risk of breast cancer after immigration (36). We also discussed breast cancer cases with both good and poor outcomes and the importance of early detection. At the end of the educational session, we allowed participants to ask questions to minimize any panic and fear that may have occurred as a result of the discussion of breast cancer.

## Sociocultural Strategies: Making Health Information Culturally Appropriate

To provide an appropriate context and meaning to our information and messages, we sought to discuss breast cancer and its screening practices in the context of Chinese cultural values and beliefs (9,37). Qualitative research with Chinese-Australian women indicated that the concept of fatalism (the idea of "letting things be") and social stigma relating to cancer (2) are barriers to breast screening participation; barriers to undergoing breast cancer screening include cultural taboos about touching one's body and a sense of modesty about exposing the breasts to examination (2,38,39).

To address the participants' fatalistic views, we applied the principle of *karma*, which encourages people to do "good" and "right" and receive "good" in return. The key message was that the earlier cancer is detected, the better the treatment outcome. We confronted the issue of social stigma by emphasizing that the breast is merely an organ of the body, that breast cancer is not related to past sexual activities, and that talking about breast cancer is not shameful. Furthermore, we emphasized that cancer, including breast cancer, is in no way contagious. For example, we presented a scenario showing that there is no risk of contagion when dining with cancer survivors. Finally, we addressed cultural taboos relating to modesty and touching one's body by reassuring participants that the technicians operating the mammography equipment are all female and receive clear instructions to minimize unnecessary body contact during the procedure.

## Linguistic Strategies: Ensuring Effective and Culturally Appropriate Communication

Making materials linguistically accessible can include presenting materials in participants' native language(s) and in accordance with cultural norms and values (9). Because our participants were Cantonese speakers, we presented all program information in Cantonese and used simple wording throughout. To ensure that the materials were culturally appropriate and that the proper meaning and context were given to translated materials, all program information was double-checked by 3 people who shared the same cultural and linguistic heritage as the participants.

Third-person statements were used to avoid a sense of confrontation and to maintain social harmony. For example, rather than saying, "If you have smaller breasts, you have less chance of getting breast cancer," we said "If a woman has smaller breasts, she has less chance of getting breast cancer." We also used metaphors in our education session and materials, consistent with typical Chinese communication patterns in which "meaning lies beyond words" (4,40). In Chinese communication, meaning deepens as one engages further in comprehension and contemplation (40), and metaphors are used frequently for discussing health-related matters. Using metaphors also helped us avoid the use of direct, confrontational terms relating to sickness and helped minimize discomfort for participants.

We presented practical tips for having mammograms to increase motivation to participate in breast screening. For example, we explained how to make an appointment and check on the availability of interpreters and provided a demonstration of the actual procedure to reduce participants' worries about language barriers.

## Constituent-Involving Strategies: Incorporating the Experience of Members of the Chinese Community

Involving members of the community in intervention planning and decision making is an important strategy (9), and we used 2 approaches to involve community members in LWHB. First, we worked with a Chinese female volunteer and members of 2 Chinese community organizations throughout the program planning and design process to ensure culturally appropriate program content. Three members from these organizations shared with the research team the cancer experience of their family or friends, which was incorporated into program content, and 1 of the community members was also a participant in the program.

Second, we engaged program participants as lay health advocates, a common constituent-involving strategy (9). Participants received training to serve as breast health advocates and disseminate messages about breast health in their communities; this training was integrated into the educational program. We used role-playing exercises to help participants feel comfortable in this role. These scenarios aimed to improve participants' understanding of breast health and give them an opportunity to practice communicating the breast health message to many different women, including grandmothers, mothers, peers, and younger women. This training session also provided an opportunity for participants to share the cancer experience of their family or friends with the role-play group members. At the end of the session, we allowed participants to share their concerns and tips with one another to solicit feedback and build participant confidence.

## Challenges and Implications for Practitioners

We encountered challenges during the implementation of LWHB. First, considerable time was required to ensure that the program was culturally appropriate. Developing the information kits and other printed materials alone took 2 months, and 5 meetings were conducted with the organizations and 10 with the research team during the design and consultation process. Practitioners should have a realistic timeframe when planning and developing such programs. Furthermore, the program was designed for a Cantonese-speaking audience. Modifications may be required if the program is delivered to audiences who speak Mandarin or other Chinese dialects.

## Conclusion

Five commonly used strategies for targeting health messages to different populations were employed to design and implement LWHB for Cantonese-speaking Chinese immigrant women in Australia. Program development and implementation required careful assessment of wording, color choice, location, and communication patterns, and required addressing barriers (eg, modesty). Grounding LWHB in a framework of traditional Chinese philosophies and delivering health messages in a context of cultural beliefs provides a useful blueprint for public health practitioners interested in working with Chinese immigrant communities. Modifications may be necessary if the program targets women who speak dialects other than Cantonese. Practitioners should seek out community leaders to obtain advice on modifying the program to tailor it to selected community members.
